# Nursing guidelines and multidisciplinary approach of chronic pain

**DOI:** 10.1590/1518-8345.0000.4017

**Published:** 2023-11-03

**Authors:** César Calvo-Lobo

**Affiliations:** 1 Universidad Complutense de Madrid, Facultad de Enfermería, Fisioterapia y Podología, Madrid, España.

**Figure:**
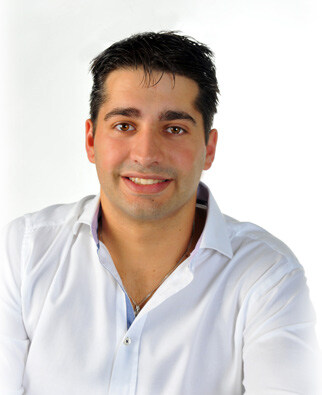


 Worldwide, chronic pain supposed a global burden condition with high associated morbidity and disability^(^ 1 ^)^. Nurses were considered as one of the healthcare providers in common face-to-face contact with patients who suffered from chronic pain. Thus, pharmacologic management of chronic pain may be facilitated by nurse practitioners in conjunction with the medical staff avoiding prescription barriers and emphasizing inter-professional collaboration and patient education. These guidelines to identify facilitators and barriers for chronic pain management by nurse practitioners could improve chronic pain interventions by nursing management in the healthcare system^(^ 2 ^)^.

Chronic pain may be defined as recurrent or persistent soreness for more than 3 months which represented a key healthcare problem affecting up to 19% of the European population and 20% of the American population. This condition implicated not only a temporal extension of acute pain, due to it comprised physiological nociception mechanism as well as pathogenetical and physical alterations, such as central sensitization linked to altered modulation of pain disorders, activation of glial cells and modified processing of neuroimmune signaling. This chronic process may be also influenced by different social and psychological factors, including pain catastrophizing, depression, fear avoidance behaviors, somatization processes, as well as different cultural attitudes. Indeed, a biopsychosocial approach of patients who suffered from chronic pain should include new strategies for prevention, evaluation and management. Chronic pain was used as an umbrella term including a wide range of clinical alterations, such as migraine, fibromyalgia, chronic fatigue or long musculoskeletal pain disorders without a specific cause. Therefore, biopsychosocial factors linked to chronic pain disorders played a key role in the maintenance and chronification of these disorders. The implementation of new educational strategies for healthcare providers and patients suffering from chronic pain about biopsychosocial approach assessment, prevention and care should be prioritized^(^ 3 ^)^.

Chronic pain conditions, such as fibromyalgia, involved physical and psychological factors which should be considered during nursing policy, research, practice, management and education^(^ 4 ^)^. Chronic conditions, being pain considered as one of the key focus, were presented as one of the most relevant priorities for nursing guidelines especially in primary health care because nurse practitioners were first-line healthcare providers with face-to-face contact with patients who suffered from chronic pain. Multidisciplinary interventions were more beneficial than isolated disciplinary treatments in order to promote health and prevent chronic pain conditions in primary healthcare. This multidisciplinary approach should include different healthcare specialist such as medical staff, physical therapists, occupational therapists, psychologists and, among others, nurse practitioners. Multidisciplinary treatments leading by nurse practitioners provided strong effects in primary healthcare environments, shortening the length of stay of patients in hospitals, decreasing complications and minimizing anxiety and depression levels. In addition, these multidisciplinary interventions promoted the self-management ability and quality of life of patients with chronic conditions, painful diseases among others^(^ 5 ^)^.

Thus, nursing policy, education and management in order to improve the morbidity and disability, linked to chronic pain conditions^(^ 1 ^)^, should experiment a rapid evolution implementing new guidelines for healthcare systems^(^ 2 ^)^, especially organizing the primary healthcare of first-line practitioners such as nurses and physical therapists in conjunction with medical staff, psychologists and other healthcare providers^(^ 5 ^)^. The complexity of chronic pain mechanism and features should be well-known by nurses and healthcare providers due to this label may be not only a medical diagnosis, including a wide range of psychological, social and biological alterations which required biopsychosocial approaches leaded by multidisciplinary interventions^(^ 3 ^)^.

In conclusion, nursing guidelines should include novel guidelines for policy, research, practice, management and education in accordance with multidisciplinary approaches in order to improve complex chronic pain conditions according to the biopsychosocial model.
